# Integrating a Sport-Based Trauma-Sensitive Program in a National Youth-Serving Organization

**DOI:** 10.1007/s10560-021-00776-7

**Published:** 2021-06-05

**Authors:** M. Shaikh, C. Bean, L. Bergholz, M. Rojas, M. Ali, T. Forneris

**Affiliations:** 1 University of Ottawa, Ottawa, ON Canada; 2 Brock University, St. Catharines, ON Canada; 3 Edgework Consulting, Boston, MA USA; 4 BGC Canada, Toronto, ON Canada; 5 The University of British Columbia, Kelowna, BC Canada

**Keywords:** Community-engaged, Child, Knowledge translation, Evaluation, Scale-out

## Abstract

There is a pressing need to equip youth-serving community organizations to respond to the unique needs of trauma-exposed children. Early prevention measures can be an effective means of redirecting children to self-regulatory healing, while facilitating their transition toward strength-based thriving. Sport can offer a powerful opportunity to reach these children; however there remains little information on how to effectively develop, deliver, evaluate, and sustain trauma-sensitive sport programs in a community context. The purpose of this paper is to outline a case study of integrating sport-based trauma-sensitive practices with BGC Canada’s national Bounce Back League program. An interdisciplinary partnership of academic, community, and practice experts used a community-based participatory action research approach, paired with a knowledge translational approach, to guide the process of program development. Mixed methods (e.g., surveys, logbooks, interviews, focus groups, online communications) were used to generate ongoing insights of staff’s training experiences, successes and challenges of program implementation, and potential impact of program on club members. Several stages of program development are described, including: (a) collaboratively planning the program; (b) piloting the program to three clubs; (c) adapting the program using pilot insights; (d) expanding the adapted program to ten clubs; and (e) creating opportunities to maintain, sustain, and scale-out practices throughout grant duration and beyond. Lessons learned regarding the leadership team’s experiences in terms of developing, adapting, and integrating trauma-sensitive practices in this community context are shared.

**Supplementary Information:**

The online version contains supplementary material available at 10.1007/s10560-021-00776-7.

The high prevalence of traumatic exposure in childhood underscores the need for systematic approaches to mitigate potential harm (Felitti et al., 2019). In Canada, 32% of adults report that they experienced abuse in childhood (Afifi et al., 2014). Childhood maltreatment has been associated with increased risk of negative outcomes in adulthood (e.g., depression, anxiety, psychosis, and suicidal behaviors; Li et al., 2020; Liu et al., 2017). Several different childhood experiences can contribute to traumatic consequences, including abuse, neglect, household dysfunction, peer victimization, racial discrimination, and community violence exposure (Alvarez, 2020; Matheson et al., 2019). Children living in under-served communities (e.g., from families facing inadequate housing conditions, financial strain, or food insecurity) are disproportionately exposed to and affected by traumatic experiences (Drake & Jonson-Reid, 2014). Community workers can use a strengths-based approach to mitigate these negative consequences and foster psychosocial skills in children, including resilience (i.e., positive adaptation in the face of adversities; Folke, 2016); trauma-sensitive practices have been gaining traction as an effective means to promote resilience-building.

Trauma-sensitive practice involves leveraging protective factors that promote healthy development in child engagement activities (e.g., supportive adult–child relationships, sense of safety, opportunities to build competence; NCTSN, 2015). The Substance Abuse and Mental Health Services Administration (SAMHSA, 2014) proposed four “Rs’ of trauma-sensitive practice: (a) realizing the widespread impact of trauma, (b) recognizing the signs and symptoms of trauma in children, (c) responding by embedding trauma knowledge into practice and policy, and (d) proactively resisting re-traumatization. Community workers routinely encounter trauma-exposed children (Mersky et al., 2019); when these workers integrate trauma-sensitive practice, they are more responsive to these children’s needs and are better equipped to intervene and reduce trauma-related harm. The evidence base on trauma-sensitive practice is growing; initiatives in early childcare and education, schools, child welfare, and behavioral health, have observed improvements in positive developmental outcomes for children (e.g., Bartlett et al., 2018) and reduction in children’s trauma-related symptomatology (e.g., Barto et al., 2018).

## Trauma-Sensitive Sport

Community sport contexts offer popular, enjoyable, and motivating opportunities to build skills (Holt et al., 2020), and can reach many unscreened trauma-exposed youth (Bergholz et al., 2016). For trauma-exposed populations, sport can offer healthy psychological escape, sense of belonging, and promote embodiment and control (Massey & Williams, 2020). However, sport can also expose children to negative experiences (e.g., bullying, maladaptive coaching practices, overwhelming pressure from ego-oriented climates; Crane & Temple, 2015). These experiences may be especially salient for children from under-resourced communities, and can trigger re-traumatization (Massey & Williams, 2020). Intentionally structured sport can be an avenue to mitigate negative experiences while promoting positive outcomes (Bean & Forneris, 2016).

Trauma-sensitive sport programs adopt a holistic model to youth development in using both deficit-reduction approaches (i.e., philosophies on how intentional youth engagement can mitigate trauma-related consequences) and strength-based approaches (e.g., ways of thinking in which all youth can thrive). In trauma-sensitive sport programs, staff are trained on the negative impacts of trauma (e.g., emotional dysregulation, impairments in social relatedness), how trauma symptoms may be exhibited in sport (e.g., irritability, lack of focus, peer conflict), how sport can be used to build children’s resilience from traumatic exposure (e.g., motivating environments, caring child–adult interactions), and how to leverage and re-design sport (in terms of playing spaces, equipment, rules, roles, and structures) to support children’s resilience building (Bergholz et al., 2016). However, the use and assessment of such initiatives in sport settings is limited.

There is a dearth of literature regarding the processes of integrating trauma-sensitive practices within community sport programs. Often, literature on sport-based youth development programs focus on detailing program outcomes, without also detailing implementation processes (i.e., how the program was developed, the program content, and how it was delivered; Holt et al., 2020). Such work can unearth context-specific experiences of integrating these practices (i.e., successes and challenges), which in turn can help inform best practices for improvement and sustainability of youth sport programs. Therefore, the purpose of this paper is to: (a) describe a case study of the Bounce Back League (BBL), a trauma-sensitive sport program hosted in multiple clubs of BGC Canada (formerly known as Boys & Girls Clubs of Canada), a national non-profit youth-serving community organization; and (b) outline lessons learned in the development of this program. The authors used a knowledge translation (KT) approach to describe how evidence-informed, sport-based, trauma-sensitive practices were translated and adapted to the BGC Canada community context, and how these practices were maintained and sustained.

## Context and Partnership

Collaborative and mutually supportive relationships between community programmers and scholars offer multiple avenues of expertise in informing design, delivery, monitoring, and evaluation of sport-based youth development programs, and can promote program sustainability (Coakley, 2011; Welty Peachey et al., 2018). In 2016, the BBL project emerged from an inter-disciplinary partnership between three researchers, two expert trauma-sensitive sport consultants, two BGC Canada administrators, and managers from three BGC Canada clubs. This partnership, known as the BBL leadership team, collaborated on decisions and activities across all phases of BBL program development (i.e., conceptualization, planning, training, implementation, evaluation, adaptation, maintenance, and sustainability). The leadership team used a community-based participatory action research (CBPR) approach; here, all team members were equitably involved to produce actionable change in improving a community-identified issue (Coughlin et al., 2017). This research was grounded in pragmatic epistemological perspectives, where people and their contexts are in constant interaction, and these interactions shape the environment and the knowledge that is generated; thus, the research design was adaptable based on ongoing reflection and collaborative decisions made by the research team to address a shared goal (Hall, 2013).

## Guiding Approach: The Knowledge-to-Action Cycle

To understand and guide the use of knowledge in practice, it is useful to refer to KT approaches; knowledge translation involves the exchange, synthesis, and application of knowledge through a dynamic and iterative process of interactions between relevant stakeholders (Bowen & Graham, 2013). Using KT approaches within community partnerships are ideal for augmenting the uptake, integration, and sustainability of community programs (e.g., Bean, Rocchi, et al., 2020; Bean, Sewell, et al., 2020). The knowledge-to-action cycle (KTAC; Graham & Tetroe, 2010) can help visualize the process in which sport-based trauma-sensitive practice was actioned in BGC Canada. The KTAC has been recently used to guide studies in sport-based youth development (e.g., Holt et al., 2018) and trauma-related practices (e.g., Pizzirani et al., 2020). Two components comprise the KTAC (*knowledge creation* and the *action cycle*). *Knowledge creation* involves synthesizing primary studies to inform and develop knowledge products that can be actioned by knowledge users. The *action cycle* concerns the use of these knowledge products and spans seven phases: (a) identifying the problem; determining the know-do gap; and identifying, reviewing, and selecting knowledge relevant to the problems or gaps; (b) adapting selected knowledge to the context in which it will be used; (c) assessing barriers and facilitators to knowledge use in the local context; (d) selecting, tailoring, and implementing interventions to enhance knowledge use by potential knowledge users; (e) monitoring knowledge use; (f) evaluating outcomes of knowledge use; and (g) sustaining knowledge use. The application of KTAC can be iterative, with fluid boundaries between phases and reciprocal loops where practices in one phase can inform practices in other phases (Graham & Tetroe, 2010). Such was the case in this project.

## Mobilizing Knowledge into Action in the Bounce Back League

**Fig. 1:**
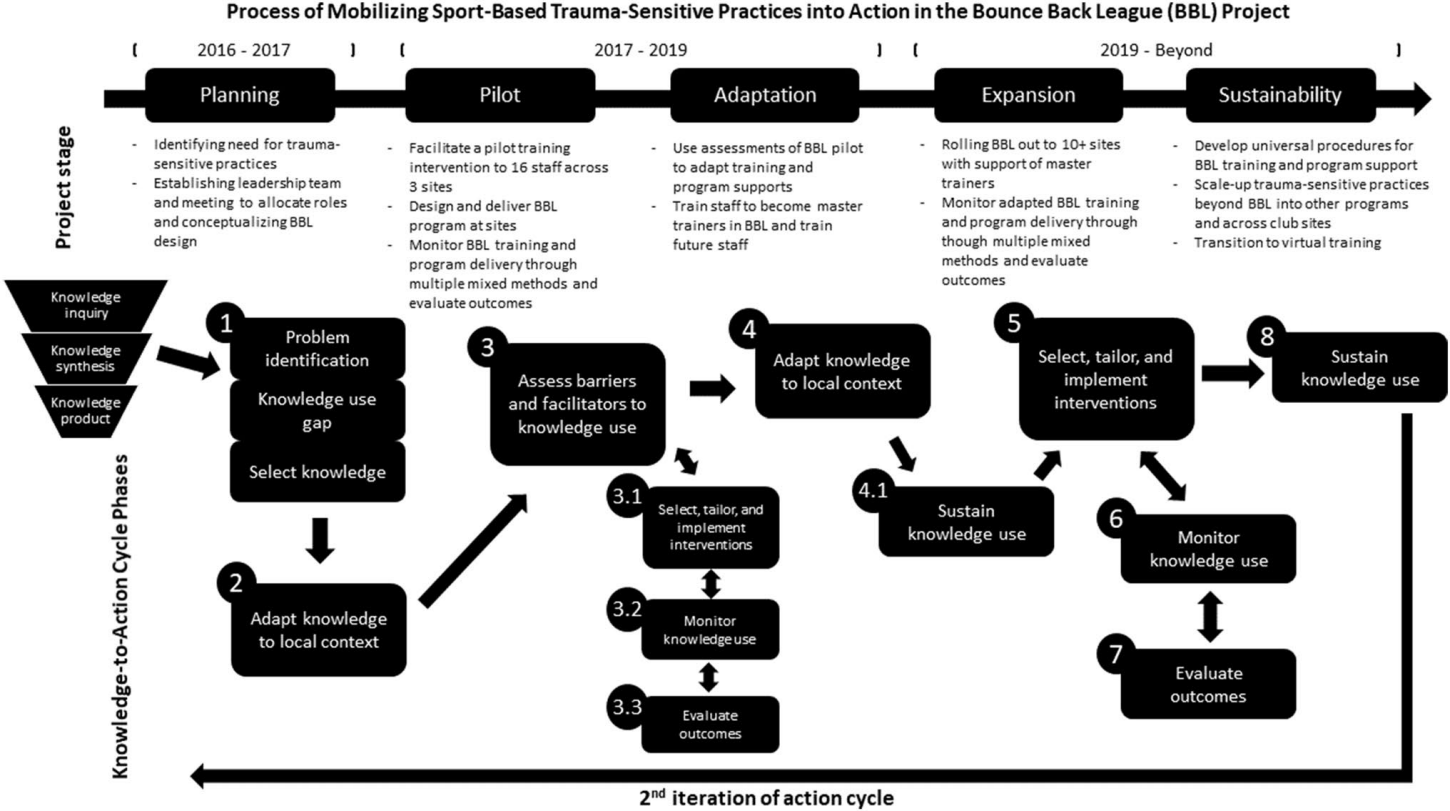
Process of mobilizing sport-based trauma-sensitive practices into action in the bounce back league (BBL) project

A case study methodology was used to understand the phenomenon of translating sport-based trauma-sensitive practices to a community context (Creswell & Poth, 2018). This case study is bounded to the BBL program, developed by the leadership team, and hosted in 18 BGC Canada clubs, involving 80 + club staff and 600 + members. The KTAC guided project development and followed five stages: (a) planning—problem-identification and conceptualizing BBL to align with BGC Canada’s needs; (b) the pilot—BBL rollout across three core clubs; (c) adaptation—using pilot insights to adapt BBL; (d) expansion—BBL rollout across 10 additional clubs; and (e) sustainability—capacity-building and knowledge dissemination activities to improve BBL and trauma-sensitive practices in BGC Canada (see Fig. 1 for the interactions between BBL stages and KTAC phases).

### Planning

In the planning stage, the KTAC phase of *identifying a problem, determining the know-do gap,* and *identifying, reviewing, and selecting knowledge,* were focused on. The leadership team identified the *problem* that many BGC Canada club members (hereafter referred to as ‘members’) face risk of traumatic exposure (e.g., > 70% of the families served are in low-income situations). These members may lack access to health promotional supports, especially if they have not been screened with a trauma-related disorder, despite exhibiting trauma-related symptoms. As well, many members may not have the developmental capacities to understand or articulate their experiences of trauma—or lack power to disclose when the sources of trauma are from their close relationships. Thus, the leadership team identified the need to equip BGC Canada staff to support members’ unique needs. Many staff were already equipped with training related to facilitating positive youth development (e.g., HIGH FIVE®), mental health first-aid, crisis intervention, and referral protocols when identifying children who needed external health supports or clinical care. As well, many staff possessed experience in facilitating several youth developments programs across domains of leadership, education, creative arts, and recreation. However, many staff were not equipped with knowledge (*know*) or skills (*do*) to support trauma-exposed members in times of dysregulation, promote self-regulation practices, or design environments that minimize harm and foster resilience-building.

To address this *know-do* gap, the knowledge that was *identified, reviewed, and selected* was sport-based trauma-sensitive practices and the purpose of this project was to build BGC Canada staff’s application of these practices. These practices were chosen given the popularity of BGC Canada’s sport programs, the numerous benefits these practices offer (Massey & Williams, 2020), and the ease to which these practices can be embedded into staff’s existing work. Trauma-sensitive practices also operate at a low-profile—programs do not need to be marketed as trauma-related to members or caregivers; rather, programs can be branded as unique sport experiences that intentionally teach resilience-related life skills through sport. This strategy avoids stigmatization, allowing all members to participate, undifferentiated by their trauma.

Next, *adapt knowledge to the local context* was focused on in the BBL planning stage. This process involved adapting sport-based trauma-sensitive practices to the BGC Canada context, within their existing human resources, physical spaces, and organizational capacities. The leadership team met over three days to discuss adaptation and development. The core objectives of these meetings were to: (a) provide psychoeducation to stakeholders on sport-based trauma-sensitive practice, (b) conceptualize a program that can be adapted to BGC Canada clubs, (c) generate buy-in from key BGC Canada managers who may champion the approaches, (d) develop a plan and establish timelines for the project, (e) formulate an evaluation process and identify key outcomes, and (f) solidify a process for collaboration and communication as a leadership team.

From these meetings, the leadership team developed connections with each other, generated value and excitement for the project, and collaboratively established the project goals, which included: (a) develop a training program that will build staff’s competencies to apply sport-based trauma-sensitive practices at their clubs, (b) have staff implement these practices as the BBL program across 13 clubs (reaching 20–25 members [9–12 years old]/club, per season [3 seasons a year]), (c) use a train-the-trainer model to build BGC Canada’s capacity to maintain and sustain BBL, and (d) evaluate program components to inform BBL adaptations. In line with each goal were the intended outcomes: (a) staff implement BBL effectively at their clubs; (b) BBL members experience improvements in basic psychological needs support, resilience-related life skills, and well-being; (c) a master trainer team is formed to train future BBL staff beyond grant duration; and (d) program materials are developed with guidelines for effective BBL implementation.

The leadership team established to communicate regularly on BBL program development over monthly phone/video calls. Roles of team members were also established. Here, BGC Canada administrators were responsible for coordinating, communicating, and checking-in with clubs on an ongoing basis, which included relaying expectations and arranging for staff involvement in BBL trainings. The consultants were responsible for designing and delivering the sport-based trauma-sensitive practice training to staff and holding ongoing phone/video calls with lead staff at each club to answer design questions and offer feedback. BGC Canada managers were responsible for participating in trainings and managing their own club’s design and delivery of BBL.

The researchers were responsible for developing evaluation tools, training staff on how to use the tools, assisting clubs with collecting data during on-site visits, analyzing and communicating these data, and soliciting staff feedback regarding the feasibility of evaluation activities in practice. The program was to be carried out over 4 subsequent years, including 2 years of piloting across three clubs with ongoing adaptation. Evaluation findings from pilot and adaptation stages would inform the expansion to 10 clubs in the final 2 years. Use of a train-the-trainer model would help build and maintain project sustainability beyond grant duration.

### Pilot

The pilot stage involved implementing the BBL training with three BGC Canada clubs, who then went on to design and deliver their first seasons of BBL—with the leadership team’s close monitoring, evaluation, and ongoing support, as per their established roles in the planning stage. In alignment with the KTAC phase *assess barriers and facilitators to knowledge use*, this BBL stage focused on exploring the successes and challenges that arose in the pilot. Given that multiple KTAC phases can be an undergone concurrently, three sub-phases were also carried out: *select, tailor, and implement an intervention*, *monitor knowledge use,* and *evaluate outcomes*. A multi-faceted, sport-based, trauma-sensitive practice training intervention was designed for staff to learn how to develop BBL programs at their home clubs. Twelve staff participants (three managers, three supervisors, six coaches) across three clubs, were gathered to attend an initial 3-day intensive workshop.

The workshop was primarily facilitated by the consultants, with the support and participation of the researchers and BGC Canada administrators. The topics broadly covered: (a) psychoeducation of the prevalence and impact of trauma on the child, (b) factors that contribute to trauma healing in sport, (c) strategies to intervene with members in times of dysregulation, (d) strategies for coaching and re-designing sport to facilitate resilience-building, (e) strategies to facilitate members’ skill development. The topics were further adapted in subsequent iterations of the training workshop during the BBL’s adaptation stage (see Table 1 for list of workshop modules). The facilitators used delivery strategies such as brief lectures, story sharing, paired and group discussions, active games and sports, coaching practice, role play activities, and workbook activities. Staff received a guidebook as a resource to use post-workshop as they returned to their home clubs to implement BBL. Staff gathered again at 4- and 12-months into programming to attend refresher workshops (2-days each). An online social learning space was established via *Slack*, where the leadership team and participating clubs shared experiences, resources, strategies, media, and questions with one another throughout the project, in effort to ease access to communications, resources, and learning opportunities.

**Table 1:** Description of BGC Canada—adapted BBL training modules

#	Module	Description
1	Understanding trauma	Understanding the parts of the brain, the stress-response system and defining trauma
2	How trauma shapes behavior	Understanding adverse childhood experiences (ACEs) and their link to negative consequences, the impact of trauma on a child’s behavior, and how children’s behavior can push us away
3	How healing happens	Four steps of healing: participation, regulation, daily functioning, and better place. Identifying mechanisms in clubs and staff’s interactions that promote healing. Becoming trauma-sensitive
4	Good group management	Principles and strategies for group management to support children to participate effectively
5	Stepping in during times of dysregulation (Part I)	Identifying when a child needs more help, understanding the difference between skill and will, and helping participants develop the skill of self-regulation, through deep breathing, taking timeouts, timed practice, individualized schedules, focusing on one skill, leveraging social support in regulation, redirected questioning, and developmentally appropriate challenges
6	Stepping in during times of dysregulation (Part II)	Connecting with the dysregulated participant using the SEPR system of stabilize, explore, plan, and return. Understanding what the child is going through at each stage and what they need
7	Introduction to the Bounce Back League (BBL)	How sport can heal through several elements: home field advantage, seasons of play, competence, physical activity, team and community focus, immersion and engagement, organization and structure, decision-making, and real stakes. Introduction to BBL as a trauma-sensitive sport experience, leveraging competency, competition, team-orientation, caring coaches, physical activity, and core principles of positive youth development
8	The BBL Workout (Part I)	Introducing and describing the intent behind each component of the BBL Workout structure: arrival, warm-up, skill play, transition time, game time, cool down, team time, and departure
9	The BBL Workout (Part II)	Goals and strategies for coaching each BBL Workout component and designing a full workout. Planning a season of play across several workouts, setting rules and expectations, coordinating coaching in pairs, and evaluating the workouts and seasons of play
10	Coaching sport	Understanding how to be a caring coach, and matching different coaching styles (guided discovery, command style) to different situations
11	The BBL skills	Understanding the BBL skills (come to play, build my team, and play), their intentions, and how to integrate them into coaching
12	Championing trauma-sensitive approaches in our clubs and communities	How to share the BBL with others and build the BBL skills across programs, staff, and clubs. Using a resilience-building, strength-based focus in communications, and avoiding stigmatization

During program implementation, each club aimed to recruit 20–25 members (ages 9 to 12) per season. The goal was for each club to run three seasons a year, with each season building on the relationships and skill development of the previous season. Members were welcome to participate in multiple seasons. Clubs could choose season length (6–10 weeks), the sport (e.g., basketball, soccer, ball hockey), and session frequency (all clubs chose 1/week). All clubs were responsible for structuring sessions around a universal BBL workout structure, comprised of seven intentional components (see Table 2 for complete details): (a) arrival, (b) warm-up, (c) skill play, (d) transition time, (e) cool down, (f) team time, and (g) departure. This structure offered a predictable schedule of activities, in which members could experience safety, skill-building opportunities, and practice regulating themselves in competitive, real stakes environments (Bergholz et al., 2016).

**Table 2:** Description and suggested duration of BBL workout structure and the BBL skills

BBL Workout		
Activity	Description	Timing
Arrival	A welcoming sport environment where coaches connect with players as they transition into the sport session. Coaches reinforce feelings of belonging (e.g., greet players individually and by name), identify players who may be entering in a tough place and consider pulling them aside for a check-in	5 min
Warm-up	A structured sequence of activities to help players tune-in and center themselves in the present, elevate their heart rates, and prepare their bodies and brains to fully participate. Coaches leverage activities like practicing pulse checks, deep breathing, body scan stretches, player-led warmups, and paired activities	5 min
Skill play	1–2 specific games that prioritize: (a) *engagement*—maximize movement and fun, minimize instruction; (b) *repetition*—facilitate rhythm, rehearsal, and skill progression; (c) *choice*—players decide own pace/challenge. Coaches monitor skill play and engage in coachable moments (e.g., demonstrate a skill, reinforce praise, adjust task difficulty)	10–20 min
Transition time	Time for players to take a structured break and hydrate. Coaches can organize players into teams for Game Time	5 min
Game time	A competitive environment that exposes players to test sport and life skills under dynamic circumstances and real stress/stakes (i.e., keeping score). Sport is re-designed to optimize engagement and skill-building opportunities (e.g., small-sized playing spaces, unlimited timeouts, non-punitive referee as a coach). Coaches seek parity in team composition, enable fair playing time for all players, provide specific praise to players in gameplay while teaching/interacting with players on the bench, and intervene one-on-one with players during challenging situations	30–35 min
Cool down	A structured sequence of activities to help players regulate their breathing and heart rates and begin preparation to transition out of BBL. Coaches revisit Warm-Up activities (e.g., heart rate) and asks players to assess any changes	5 min
Team time	A carefully and creatively facilitated conversation with players and coaches intended to draw out key learnings, promote reflection and connection and help players apply what they are learning in sport to their lives. Coaches probe players to share highs and lows, give praise to one another, revisit specific in-game moments, discuss most significant learnings, ask burning questions, identify specific BBL skills used in the session or season, and discuss examples where players can use (or have used) skills with their peers, at home, at school, or in their communities	5–10 min
Departure	A time to help players transition out of the sport session safely and successfully. Coaches create informal time with players, remind players how they can transfer skills to life, and check-in with players who may be in a tough place	5 min

BBL skills			
Skill	Description	Domains	Coaching strategy
Come to play	Knowing myself and preparing to make the most of the opportunities and challenges in front of me	Engagement and readiness	Promotion of willingness, preparation, and full immersion in activities
Build my team	Investing in people to form a system for support and skill-building together	Interpersonal effectiveness	Promotion of teamwork, leadership, prosocial behaviours, interpersonal effectiveness, respect for others, peer support, and seeking support
Play on	Persisting and adapting when faced with adversity and staying on my positive path	Emotional awareness and regulation	Promotion of awareness of self, valuing rest, perseverance, overcoming challenges

Staff were trained on several techniques to proactively facilitate members’ regulation (e.g., practicing deep breathing to help calm emotions, allowing unlimited timeouts to rest when feeling overwhelmed, and structuring a space [*The Zone*] to enter, self-regulate, and return to main activities when ready). Staff also adopted a 4-step framework to connect with members in times of dysregulation: (a) stabilize—support member in a triggered state to re-regulate and arrive to a place where they can have a conversation, (b) explore—facilitate a judgment-free inquiry about what the member is experiencing, (c) plan—co-construct strategies to resolve what happened and to regulate better in the future, and (d) return—co-construct strategies to transition back to the activities on the member’s own will.

The program also focused on teaching three BBL skills tied to resilience-building (see Table 2): (a) come to play (e.g., preparation, self-awareness), (b) build my team (e.g., empathy, seeking support), and (c) play on (e.g., effort, recovery). Strategies to facilitate these BBL skills included defining skills out loud, having a ‘skill of the week’, posters with skills and definitions, encouraging and praising skill use, and reflecting on skills during team time (Bean et al., 2018). These skills were constructed based on evidence of what works in trauma-sensitive sport programming (Edgework Consulting, 2020), understandings of life skill development through sport (Holt et al., 2020), and BGC Canada core values (BGC Canada, 2020). See Supplement 1 for strategies on facilitating BBL skills within the BBL Workout Structure.

In evaluating the program, three questions were proposed: (a) how did staff perceive their training experiences and what value was generated from these experiences? (b) what were the successes and challenges that staff met in implementing their training into practice? and (c) what was the impact of programs on members? A mixed-methods approach was used to address these questions. To explore staff’s training experiences, knowledge surveys and interviews were conducted pre- and post-training, and field notes, pictures, and audio-recordings were taken during training. To monitor successes and challenges, staff completed structured logbooks after each BBL session, participated in multiple focus groups and interviews, and shared conversations on Slack. To examine program impact, members completed surveys on well-being (Tennant et al., 2007), resilience-related skills (Windle et al., 2011), and basic needs support (Bean, Rocchi, et al., 2020; Bean, Sewell, et al., 2020). Members also took part in interviews and arts-based activities, and staff completed report cards of members’ participation (Shaikh et al. 2020a, 2020b).

Evaluation results related to staff’s training experiences revealed that many desired outcomes were reached, including high satisfaction and usefulness of the training workshop for staff, building staff’s BBL-related knowledge and skills, and staff’s use of trauma-sensitive practices in the BBL and into their everyday lives (Shaikh et al., 2018; Shaikh, Bergholz, et al., 2019b). Several facilitators to knowledge use were identified: (a) staff’s existing experiences with trauma-exposed members in BGC Canada helped ease adoption of sport-based trauma-sensitive practice, (b) staff found the training workshops highly engaging, useful, applicable, and valuable; (c) staff perceived that training content aligned with what they experienced in practice, (d) ongoing meetings with consultants facilitated continuous learning for staff, and (e) the pilot allowed room for trial and error, which eased staff’s apprehensions in applying a novel program and practice (Shaikh et al., 2018; Shaikh, Bergholz, et al., 2019b; Shaikh, Forneris, et al., 2019c).

Despite these facilitators, clubs reported several challenges (i.e., Shaikh, Bergholz, et al., 2019b) that were consistent with research in community programs (Forneris et al., 2013; Larson et al., 2015). A major difficulty was in recruiting and retaining children (e.g., scheduling difficulties, building value for the BBL, competition with other programs, referral-recruitment versus open-to-all recruitment. Inconsistent attendance made it difficult for staff to maintain an engaging experience where members can foster positive peer connections. Staff also found it challenging to balance the demands of a structured program with the myriad of children’s behavioral issues (e.g., outbursts, disengagement, aggression), given that BGC Canada staff—and members—are most familiar with unstructured, free play opportunities. As well, clubs encountered staff turnover—an inevitable reality of youth-serving organizations where many staff are part-time or in transition phases of their careers—which led to loss of capacity, especially when staff were BBL trained.

Consistent relationships are primary protective factor in supporting trauma healing (NCTSN, 2015). A benefit of having BBL managers and supervisors attend training was their capacity to step in when staff were absent. This strategy worked well given that the BBL members were also BGC Canada members who attended other programs within the club. Thus, members interacted with supervisors and managers on a regular basis beyond BBL—contributing to consistency in mentorship. Evaluation results related to program impact revealed improvements in outcomes, albeit non-significant; here, perceived well-being trended upward from pre-post season, and members who attended > 80% sessions reported higher basic psychological needs support (Shaikh & Forneris, 2020). Qualitative data analysis revealed that the members reported feeling accepted, having improved friendships, and developing sport and life skills (Shaikh & Forneris, 2020). These positive learning experiences suggested some initial effectiveness of the BBL. Overall, these evaluation findings informed BBL adaptations in the next stage.

### Adaptation

Concurrently with the pilot stage, the adaptation stage was informed by the combination of ongoing leadership team meetings, consultations and check-ins with each club, and evaluation results. The KTAC phase of *adapt knowledge to the local context* was re-visited, using insights of barriers and facilitators to knowledge use to inform ongoing BBL project development; adapting BBL processes at the organizational-level and club-level. These adaptations were communicated through email, bi-monthly meetings, Slack, and refresher training workshops. The major adaptations included: (a) tailoring the training intervention, (b) supporting staff’s motivations and accounting for staff turnover, (c) enhancing recruitment strategies, and (d) transition of training responsibilities to master trainers.

#### Tailoring the Training Intervention

The leadership team worked together to develop 12 BGC Canada-adapted modules, covering how to build staff’s awareness and value for sport-based trauma-sensitive practice, and how to structure and design a BBL program (see Table 1). These modules were the foundational structure for the training workshop and the development of BBL resources (i.e., the coach guidebook, operating manual, training manual). To address the challenges that staff encountered in intervening with dysregulated members, more focus was added to strategies for managing groups and helping members return to a stabilized state.

#### Supporting Staff’s Motivations and Accounting for Staff Turnover

At the organizational level, the leadership team established bi-monthly check-ins with all pilot clubs together. This strategy offered additional mentorship and oversight to the staff, building morale, shared understanding, and value for BBL (Ika & Donnelly, 2017), and enabled staff to share experiences and learn from each other. At the club level, managers were encouraged to: (a) choose trainee candidates that were full-time staff and planned to invest in the club long-term; (b) supervise at least half of staff’s BBL sessions and provide them feedback to help monitor, support, and enhance the quality of their BBLs; and (c) have other staff sit in on BBL sessions to observe, participate, learn, and eventually transition into BBL when faced with staff turnover.

#### Enhancing Recruitment Strategies

At the organizational level, customizable promotional materials were created (i.e., posters, infographics) with BBL branding for clubs to use in their marketing. To enhance recruitment, all clubs were encouraged to remove limits to BBL membership and invite all children to participate regardless of pre-identified risk status. This strategy helped maximize reach and inclusiveness to unscreened trauma-exposed children.

#### Transitioning of Training Responsibilities to Master Trainers

To promote the self-sustaining capacity of the BBL, a train-the-trainer model was used to train BGC Canada managers and other key staff to become BBL master trainers (Shaikh, Bean, et al., 2019a). To carry this out, an intensive training workshop was facilitated by the leadership team, in which BGC Canada managers learned and delivered the 12 BGC Canada-adapted BBL training modules to their peers and received feedback to improve their delivery. Following the workshop, these newly trained master trainers returned to their home clubs to plan the delivery of BBL training workshops to their staff. These workshops were also observed by leadership team members, who provided feedback. The master trainers were then expected to be help train newly onboarded staff in the expanded rollout.

### Expansion

The expansion involved rolling out, monitoring, and evaluating the adapted training intervention and BBL programs in ten additional clubs across Canada. Three KTAC phases of *select, tailor, and implementing interventions*, *monitor knowledge use,* and *evaluate outcomes* were undergone (again). Here, the master trainers carried out the training intervention as an intensive, in-person workshop to 20 staff, covering the 12 BGC Canada-adapted training modules. The leadership team supported master trainers in communicating expectations, coordinating travel and logistics for clubs, and modifying evaluation tools. The newly trained staff went on to deliver programs at their home clubs, while continuing to receive support from master trainers (e.g., bi-monthly calls), and the leadership team (e.g., via Slack, email, and on-site visits). The same evaluation questions were used to monitor BBL training and program implementation, using a slightly modified mixed-methods approach. For instance, items on knowledge assessments and logbooks were matched to the adapted training modules, and the length and wording of member’s surveys were simplified.

Each club ran one season (~ 6–10 weeks); the second season was cut short due to the COVID-19 pandemic. Currently, evaluation is still ongoing, as clubs offer either in-person, physically distanced program delivery, or virtual delivery. However, initial outcomes of the BBL expansion have indicated that: (a) the training intervention was successful in improving knowledge and attitudes of participating staff in sport-based trauma-sensitive practice; (b) the training intervention was perceived as enjoyable and useful for staff; (c) in most clubs, recruitment of members successfully reached 20–25 + members; however, at least two clubs were well under this mark; (d) having trained staff is essential for running high-quality BBL programs; yet, with staff turnover, clubs without trained staff resorted to traditional practices of youth engagement in sport, resulting in loss of trauma-sensitive practice. Thus, there is a need for incorporating alternative sustainability practices within BGC Canada to maintain BBL quality during and beyond grant duration.

### Sustainability

The sustainability stage involves initiatives put in place to continue BBL beyond grant- end, in alignment with the *sustain outcomes* phase of KTAC. The first goal in this plan was to maintain and expand the BBL (as a sport-based trauma-sensitive practice program) to more clubs across Canada. A second, broader goal was to build staff’s capacities in trauma-sensitive practices across BGC Canada clubs and programs, beyond sport. This second goal was constructed based on advocacy from BBL staff of the value of trauma-sensitive practices and their applicability in club work outside the BBL, as well as interest from additional clubhouses in building their club’s trauma-sensitive capacity. Several processes will contribute to reaching these two goals: (a) using a train-the-trainer model, (b) developing context-specific resources, (c) expanding to reach Canadian newcomers, (d) transitioning to a virtual platform in COVID-19.

#### Using a Train-the-Trainer Model

Using a train-the-trainer model helped build the organization’s ownership of the program, promoting internal sustainability (Shaikh, Bean, et al., 2019a; Whitley et al., 2015). Here, BBL managers who delivered BBL in the pilot were offered training to develop into master trainers and went on to facilitate BBL training for staff within and beyond their clubs. Clubs with access to master trainers were then able to host sport-based trauma-sensitive practice as a core part of their club’s training. Master trainers also train staff at a national level, whether through facilitating training workshops or consultations with newly onboarded BBL clubs.

#### Developing Context-Specific Resources

Developing context-specific knowledge products was important for communicating knowledge in clear and user-friendly ways, which can facilitate continued use of knowledge in practice (Holt et al., 2018). Materials were created that can be shared and used universally across the clubs, including the coach guidebook, training manual, promotional materials, and evaluation activities. As well, evaluation findings were shared throughout the project through user-friendly media (i.e., presentations, reports, infographics, and posters). Blogging was used as an effective means of disseminating knowledge beyond academic audiences (Stoneham & Kite, 2017); here, the leadership team published a blog on strategies for integrating trauma-sensitive practices into play (Shaikh et al. 2020a, 2020b. The authors also shared their experiences on integrating evaluation activities within community organizations along with effectives strategies for sport stakeholders to build evaluation capacity (Shaikh et al. 2020a, 2020b).

#### Expansion to Reach Canadian Newcomers

Given the importance of funding in maintaining relationships and capacity building (Coppola & McHugh, 2018), various means of extending BBL funding have been explored. BGC Canada secured funding to extend BBL across five additional clubs to target Canadian newcomer populations. Direct support for newcomers is needed as they may face unique traumatic exposure; refugees from conflict regions can face loss, grief, family separation, mental health disorders, and migratory stressors (Ley & Mario, 2019). In this expansion, an advisory group was created with BBL managers to share and co-construct strategies for meeting newcomer needs and integrating equity, diversity, and inclusion principles in BBL design, recruitment, and practice. Development of BBL for newcomers is currently underway. This extension has taken on a unique look post-pandemic, where clubs have had to shift their BBL programs to virtual and physically distanced settings.

#### Transitioning to a Virtual Platform in COVID-19

To effectively meet BBL staff’s needs in the pandemic, staff perspectives were sought through virtual meetings and a needs assessment survey. Staff largely requested more training in trauma-sensitive practices, support to shift BBL virtually, and strategies to facilitate small group, in-person activities. Thus, the leadership team delivered virtual training for staff during Summer 2020, which covered refreshers of BBL content and strategies to facilitate virtual BBL. All clubs then transitioned to a modified BBL in Fall 2020 (i.e., physically-distanced in-person or virtually). Monitoring and evaluation are currently ongoing, to gain insights of successes and challenges of facilitating BBL in a COVID world.

The abrupt shift, the need for trauma-sensitive practices across clubs in Canada, and the success in adapting the BBL based on changing conditions, made it ideal for the leadership team to pursue further options for project scale-out (Ika & Donnelly, 2017). Thus, funding was secured to develop virtual trauma-sensitive training for scale-out to BGC Canada’s 775 clubs nationally. Here, the KTAC will be re-cycled as a second iteration. The *problem identified* was the need for integrating trauma-sensitive practices across all BGC Canada clubs; the *selected knowledge* was the BGC Canada-adapted trauma-sensitive practice modules and program materials; this knowledge will be *adapted* from in-person to virtual via BGC Canada’s *virtual e-learning platform*; and this training will be *implemented* to ten BGC Canada clubs, reaching ~ 150 staff.

## Lessons Learned

The strength of the BBL partnership has led to many successes and effective responses to challenges in the BBL development process. Three lessons are shared that were most pertinent to advancing KT processes in BGC Canada. These lessons may be considered by sport, community, and recreation groups when integrating trauma-sensitive practices for effective project development: (a) meaningful change starts with small steps, (b) maintain ongoing two-way communication with stakeholders, and (c) integrate-with rather than add-on to existing activities.

### Meaningful Change Starts with Small Steps

The leadership team avoided using an all-at-once approach in both the training and implementation to ease integration and prevent overburdening staff (Bromley et al., 2015). Instead, a developmental approach was taken, involving short, intensive training workshops and ongoing consultations over a long period of time. This approach enabled more time for staff skill-building (i.e., BBL design and delivery) and learning through trial and error (an oft-preferred source of learning in sport; Walker et al., 2018). The ongoing consultations helped monitor skill development and generate insights on *high impact behaviors*—staff skills used to generate effective results. High impact behaviors are not necessarily known from the outset—their effectiveness will depend on the staff’s characteristics, the demands of the context, and the member’s needs (Larson et al., 2015). Thus, it was worth investing time in the design and pilot process to determine these high impact behaviors and prioritize them in project development. For instance, a high impact behavior in BBL was *integrating BBL skills* (e.g., naming and defining skills, using skills in informal conversations, integrating a “skill of the week”).

When staff integrated BBL skills, members were more likely to understand and internalize the skills. While effectiveness of explicit life skill teaching is supported in youth sport research (Bean et al., 2018), the developmental approach used in BBL was necessary to explore what these behaviors looked like, under what conditions do they manifest, and how effective they were (Holt et al., 2020). Then, these skills were emphasized in further training and communications (e.g., sharing strategies), and in evaluation (e.g., logbooks included a space for staff to describe how they embedded BBL skills).

### Maintain Ongoing Two-Way Communication with Stakeholders

It was beneficial to engage BGC Canada stakeholders (i.e., program managers, club staff) from the outset, and communicate using easy channels of access (i.e., regular phone/video meetings, consultations with specific clubs, emails, and Slack). This ongoing communication aligned with the project’s CBPR approach and was conducive to relationship building between the leadership team, stakeholders, and their peers (Coppola & McHugh, 2018). Such relationship building can contribute to synergies between groups and build stakeholders’ value for the project and its future (Schinke et al., 2013). Stakeholder input (e.g., feedback) and insights (e.g., club-specific successes and challenges) were also gathered in-depth through ongoing evaluation activities (e.g., interviews, focus groups, logbooks). These findings were valuable in actively supporting adaptations to existing programs, as opposed to exclusively at the end of the grant period (Brush et al., 2020). As well, open communication helped identify which clubs needed extra support and intervene with them (e.g., if clubs faced challenges in recruitment, planning activities for a wide age-range of members or integrating evaluation within a fixed time schedule).

### Integrate-with Rather than Add-on to Existing Activities

Given the multi-faceted nature of staff’s work within a community program context (Newman et al., 2020), it was important to work within the existing capacities of clubs and staff, rather than starting from scratch. For example, several clubs found it challenging to create an entirely new BBL (e.g., recruitment of members and staff, scheduling) and found that adapting an existing sport program to BBL was more feasible. Moreover, evaluation activities were developed and adapted through trial and error of what works. This process revealed that the act of *doing* evaluation was beneficial for participants.

With staff, logbooks were used to report session-by-session experiences, which brought insights into program fidelity; the logbooks also mutually benefitted staff in reflecting on the program session together—by discussing successes and challenges and setting goals for the next session. With members, quantitative pre-post surveys were used—which members found unengaging. Thus, evaluation was shifted to more creative, engaging, and participatory activities (e.g., drawing, storytelling) that could be integrated within existing programming (Shaikh et al. 2020a, 2020b). These activities enabled participants to share their experiences—which mutually benefitted the program and members in applying BBL skills (e.g., applying *come to play* by being willing to share, applying *build my team* when giving praise to another member; Jacquez et al., 2013).

## Conclusion

This case study described the process of mobilizing sport-based trauma-sensitive practices in a national community program, accomplished through a partnership between academic, community, and practice experts. For this partnership, it was essential to maintain open, ongoing collaboration in order to: (a) reinforce a shared vision, (b) co-construct insights of barriers, facilitators, and potential impacts, (c) use insights to develop high-quality adaptations, and (d) build towards sustainability, expansion, and scale-out. The success of this project helped train 80 + staff across Canada, reach 600 + members, and build increased awareness of—and need for—integrating trauma-sensitive practices across all BGC Canada clubs nationally beyond the BBL. The story of developing the BBL can offer foundational understandings of best practices in integrating trauma-sensitive practices in this community organization, which may be useful for informing intentionally planned sport in similar youth-serving groups and organizations.

## Supplementary Information

Below is the link to the electronic supplementary material.

Supplementary Information (PDF 597 KB)

## Data Availability

The datasets generated during and/or analyzed during the current study are not publicly available given the sensitivity of identifying information tied to the dataset. However, data can be made available from the corresponding author on reasonable request.
