# Supplementary material for: Integrating a Sport-Based Trauma-Sensitive Program in a National Youth-Serving Organization
Source: Child Adolesc Social Work J. 2021 Jun 5;38(4):449–61. doi: 10.1007/s10560-021-00776-7 (PMC8179094; doi:10.1007/s10560-021-00776-7)
Supplement: Supplementary file 1 — Supplementary Information (PDF 597 KB) [file 10560_2021_776_MOESM1_ESM.pdf]

The BBL Skills Cycle

Our goal is to teach these three skills, first as independent techniques and second, as three parts of an important cycle. This cycle is crucial in sport and also in life.

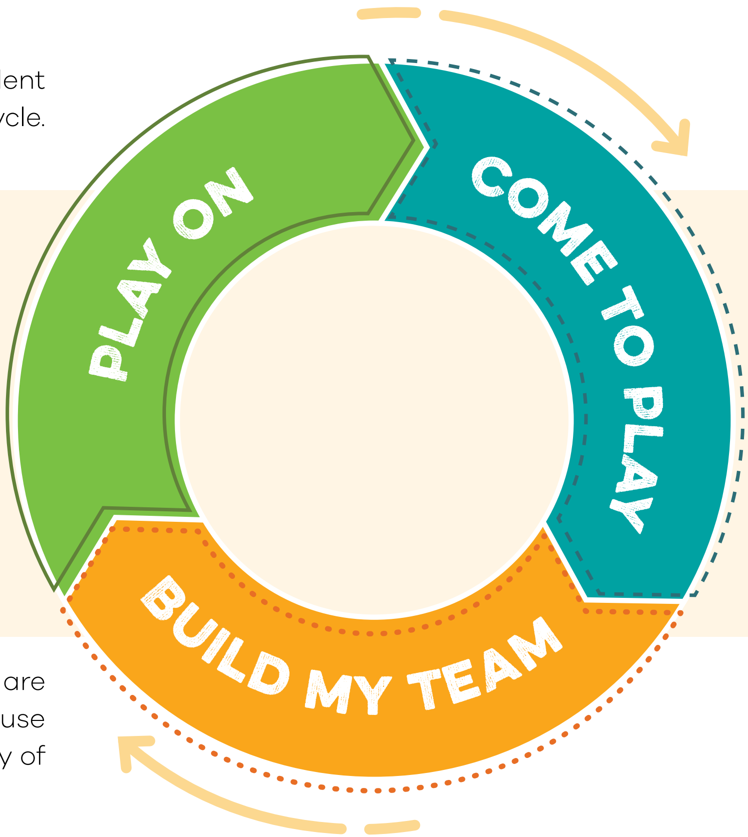

AS A PLAYER, YOU:

1. Get ready to do your best **(Come To Play)**.
2. You work with your team to try to make progress together **(Build My Team)**.
3. As you face obstacles and challenges you then adapt, adjust and persist **(Play On)**.

THEN, YOU GO THROUGH THIS CYCLE AGAIN (AND AGAIN).

By setting up the three ***Bounce-Back Skills*** as a cycle, we are able to equip players with a short sequence of skills to use to manage the dynamic and often unpredictable reality of sport and life's ups and downs.

Applying the Bounce-Back Skills

Our aspiration is for coaches to become skilled in weaving the Bounce-Back Skills into all aspects of each Workout. The table below describes several examples of places in a Workout where a Bounce-Back Skill could be used.

WORKOUT ACTIVITY	COME TO PLAY	BUILD MY TEAM	PLAY ON
ARRIVAL	This arrival time is a great opportunity to make sure you come to play. Take a few minutes to get yourself ready to make the most of this Workout.	Sit with a teammate, check-in with them. It's a great time to reconnect and build my team.	If you're coming into this Workout feeling down or stressed, try to leave it outside our Workout. You've got a chance to reset and play on here for the next hour.
WARM-UP	We use warm-ups to help everyone come to play. Each move and each activity in our warm-up should help us focus and prepare our body to play.	Building my team during warm-up means moving and stretching together. We're trying to create a fun team rhythm.	If you can't keep the rhythm in warm-up, how can you play on?

CONTINUED ON NEXT PAGE

WORKOUT ACTIVITY	COME TO PLAY	BUILD MY TEAM	PLAY ON
SKILL PLAY	Before we start this game, visualize the moves you want to make. This is one of the best ways to come to play before you start an activity.	We are going to partner up for this game. Use these pairings, not just to work on the sport skill but to build your team by paying attention to how your partner works in the activity. Get to know their style and skill so you can be an even stronger team together when it comes to Game Time.	If you find yourself unfocused or making more mistakes than you want to, it's okay to stop action during the game and do what you need to be able to play on with the activity.
GAME TIME	Let's all take 10 seconds before we start the next game to be sure we are coming to play. Do what you need to do to start the game excited, focused and prepared.	Each time we make a pass or offer support or suggestions to a teammate we build our team.	It seems like you've got more bad stress than good stress right now. If you think you can handle it, you'll be going back in to play in a few minutes. Let's talk about what would help you to play on when you are out there.
COOL DOWN	In our cool down, coming to play means we shift our focus from amped up competition and high energy to making a good transition, slowing down our heart rate and movements.	Keep an eye on each other as we cool down. You may want to build your team by checking with each other on how they are doing.	Our game time is now over and we're starting to get ready to end our Workout. Use this cool down to think about the highs and lows of your Game Time and how you want to play on with what you have next after this Workout.
TEAM TIME	If you are nervous about speaking in front of your class in school, what are three ways you can come to play to better prepare yourself?	Even as children, there are adults in our lives that are on our team. Who are the types of adults we could go to if we needed help?	If you are taking a test in school and start to feel bad stress, what are three things you could do to play on?
DEPARTURE	Use the departure time to think about what you have next after our Workout. Think about what you need to do to come to play to the next thing you are doing?	We want our team to take care of each other. That is part of what build my team means. If anyone wants to go to the next activity together, make sure you ask. You can also offer to go with a teammate to their next activity.	Remember, we have a whole season together. No matter what happened today, you'll be able to play on next week.

From Understanding Trauma to Understanding Resilience: Helping Players Help Themselves

1 COME TO PLAY

Knowing myself and ***preparing*** to make the most of the opportunities and challenges in front of me.

TRAUMA-INFORMED SKILLS:

- Reflection
- Self-Awareness
- Body Work
- Focus & Concentration
- Present Mind
- Preparation

QUESTIONS TO ASK:

- What Is My Story?
- How Am I Doing?
- Am I Ready To Play?

2 BUILD MY TEAM

Investing in people to form a system for support and ***skill building together.***

TRAUMA-INFORMED SKILLS:

- Social Awareness
- Social Confidence
- Empathy
- Encouragement
- Helping Behaviours
- Coachability

QUESTIONS TO ASK:

- What Is Our Story?
- How Do We Improve?
- How Do we Support Each Other?

3 PLAY ON

Persisting and ***adapting*** when faced with adversity and ***staying on my positive path*** no matter what.

TRAUMA-INFORMED SKILLS:

- Perseverance & Effort
- Coping
- Flexible Thinking
- Recovery
- Reflection
- Future Focus

QUESTIONS TO ASK:

- What Is Going On?
- What Are My Options?
- How Can I Keep Moving Forward?
